# Baseline Circulating Activated TFH and Tissue-Like Exhausted B Cells Negatively Correlate With Meningococcal C Conjugate Vaccine Induced Antibodies in HIV-Infected Individuals

**DOI:** 10.3389/fimmu.2018.02500

**Published:** 2018-10-29

**Authors:** Lucimar Milagres, Giselle Silva, Wânia Pereira-Manfro, Ana Cristina Frota, Cristina Hofer, Bianca Ferreira, Daniela Barreto, Marcelo Figueredo, Barbara Coelho, Lucia Villela, Constantinos Petrovas, Richard Koup

**Affiliations:** ^1^Department of Microbiology Immunology and Parasitology, State University of Rio de Janeiro, Rio de Janeiro, Brazil; ^2^Immunology Laboratory, Vaccine Research Center, NIAID, NIH, Bethesda, MD, United States; ^3^Department of Pediatrics, Instituto de Puericultura e Pediatria Martagão Gesteira, Rio de Janeiro, Brazil; ^4^Preventive Medicine Department, School of Medicine, Federal University of Rio de Janeiro, Rio de Janeiro, Brazil; ^5^Department of Periodontics, State University of Rio de Janeiro, Rio de Janeiro, Brazil; ^6^Laboratory of Immunology, Pedro Ernesto University Hospital, State University of Rio de Janeiro, Rio de Janeiro, Brazil

**Keywords:** meningococcal vaccines, HIV infection, bactericidal antibodies, T follicular helper cells, exhausted B cells

## Abstract

Since 2006, meningococcal serogroup C (MenC) conjugate (MCC) vaccines have been supplied by the Brazilian government for HIV-infected children under 13 years old. For measuring protection against MenC, the serum bactericidal antibody (SBA) assay is the method of choice. The characterization of T follicular helper cells (TFH) cells has been an area of intensive study because of their significance in multiple human diseases and in vaccinology. The objective of this study was to characterize the phenotype of peripheral TFH cells and B cells and how they associated with each other and with SBA levels induced by vaccination as well as with serum cytokine levels of HIV-infected and non-infected children and adolescents. We found that CD27^−^IgD^−^CD21^−^CD38^+^ (exhausted B cells) as well as short-lived plasmablasts (CD27^+^IgD^−^CD21^−^CD38^+^) are increased in cART treated HIV patients and negatively associated with MCC vaccine induced SBA levels. Baseline frequency of activated peripheral TFH cells was a negative correlate for SBA response to MCC vaccine but positively correlated with circulating plasmablast frequency. Baseline IL4-levels positively associated with SBA response but showed a negative correlation with activated peripheral TFH cells frequency. The increased frequency of activated peripheral TFH cells found in non-responders to the vaccine implies that higher activation/differentiation of CD4 T cells within the lymph node is not necessarily associated with induction of vaccine responses.

## Introduction

Meningococcal disease is a public health problem worldwide (1). In Brazil, case fatality rates of this disease are as high as 18–20% of cases and serogroup C meningococcus (MenC) accounts for more than 80% of reported cases (2). Since 2006, meningococcal serogroup C conjugate (MCC) vaccines have been supplied by the public sector for control of outbreaks and for high-risk patients, including HIV-infected children under 13 years old (3). Currently, the immunization schedule consists of 3 injections of a MCC vaccine in the first year of life and a booster dose in adolescents aged 12–13 years (4).

We have previously shown that only 30% of Brazilian HIV-infected children and adolescents seroconverted (bactericidal antibody titers) after one dose of MCC vaccine (5). A second dose of the vaccine induced seroconversion in about 70% of HIV-infected individuals (6). Still 30% remained without protective antibody response after two doses of vaccine. Poor antibody response of vaccinees was associated with CD4 T cell activation identified through expression of CD38, HLA-DR and CCR5 (7).

Recent studies have been focused on the characterization of circulating CD4 T cells that represent counterparts of lymph node follicular helper CD4 T cells (TFH) (8, 9). Peripheral TFH cells are characterized by high expression of the CXC chemokine receptor 5 (CXCR5) while the co-expression of other surface receptors like CCR6 and CXCR3 further defines peripheral TFH subsets with different capacity for at least *in vitro* B cell help (8, 9). In studies on seasonal influenza vaccines, the frequency of ICOS^+^CXCR3^+^CXCR5^+^ peripheral TFH cells was shown to increase only transiently after vaccination (peak at day 7) (10). This kinetics seems synchronized with the emergence of influenza-specific plasmablasts and plasma cells in blood. In contrast, a study in aging HIV-infected and uninfected women, activated (expression of HLA-DR and CD38) CD4 and peripheral TFH cells was indicative of diminished influenza vaccine-induced antibody response, mediated through TNFα production and consequently impairment of peripheral TFH-induced IL-21 secretion (11, 12). Over the past decade it has become increasingly evident that many chronic human infectious diseases to which immunity is not readily established, including AIDS, malaria, HCV and TB, are associated with fundamental alterations in the composition and functionality of B cells. A common feature of these diseases appears to be a large expansion of exhausted B cells, which are qualitatively inferior in attaining immunological control of viremia and antibody production (13, 14).

A comprehensive understanding of the biology and dynamics of peripheral TFH cells and circulating B cells may be important for the establishment of cellular determinants of vaccine-induced antibody response, which may have relevance for vaccine design or a more rational use of routine vaccines in immunocompromised individuals. Here, we characterized the phenotype of circulating B cells and peripheral TFH cells and how they associated with each other and with the protective antibody response induced by vaccination (MCC) of HIV-infected and non-infected children and adolescents. Also shown are the associations of baseline blood cytokine concentrations with the frequency of peripheral TFH cells and antibody response.

## Materials and methods

### Cohorts

We conducted a prospective cohort study at the *Instituto de Puericultura e Pediatria Martagão Gesteira, Universidade Federal do Rio de Janeiro* (IPPMG/UFRJ), Rio de Janeiro, Brazil, to investigate the secoronversion rate after MCC vaccination in HIV-vertically infected 2-18 year-old children. Details of the study were previously described (5). Baseline characteristics of HIV^+^ patients are described in Table 1.

**Table 1:** Baseline characteristics of HIV^+^ patients classified as responders (≥4-fold increase in bactericidal antibody titers) or non-responders to MenC vaccination.

**Characteristic**	Responders^*^	**Non-responders**
	**(*n* = 10)**	**(*n* = 7)**
**AGE, YEARS**		
Median (Range)	13.9 (6.4–18.4)	12.3 (8.9–16.8)
**GENDER**		
Male (%)	5 (50)	5 (71.4)
Female (%)	5 (50)	2 (28.6)
Plasma HIV RNA, copies/ml^**^	229.5 (< 50–2768)	236 (< 50–587)
Nadir CD4 count, cells/μl, blood^**^	325.5 (128–807)	374 (193–1441)
CD4 count, cells/μl, blood^**^	737 (463–953)	689 (582–1730)
CD4 percentage^**^	25.5 (15–33)	32 (20–37)
CD8 percentage^**^	46 (43–60)	43 (34–53)
Length of cART (years)^**^	6.8 (4.5–11.2)	6.2 (5.5–10.7)
Age of initiation of cART (years)^**^	4.7 (0.9–8.7)	3.3 (1.6–9.3)
% of CDC Clinical Category C	3 (30%)	4 (57%)
Mean (log_2_) of SBA titer to MenC after 2 doses of MCC vaccine	7.0	0.4

* *Only one individual was not in cART*.

** *Median (IQR)*.

### Vaccination and specimen collection

Following informed consent, the cohort received one intramuscular injections of MCC vaccine (Novartis; C Polysaccharide/CRM_197_) at the recommended dose (10 μg/0.5 ml). One year later, HIV-vertically infected children received a booster dose at the recommended dose described above. HIV uninfected (HIV^−^) controls did not receive the booster dose, as per recommendation at the time of this study, in healthy children and youth, aged 1–25 years, a single MenC dose should be given (15). For HIV^+^ group, blood samples were collected before vaccination (visit 1), 1–2 months after one dose (visit 2), before booster (10–12 months after first dose, visit 3) and 1–2 months after boosting (visit 4). HIV^−^ group had blood samples collected at visit 1 and visit 2. Heparin-treated tubes or in the absence of anti-coagulant were used and processed within 3 h after the blood draw. Peripheral blood mononuclear cells (PBMC) were separated by density-gradient centrifugation over Histopaque® (Sigma, St Louis, USA) and stored in RPMI/20% fetal bovine serum/10% DMSO in liquid nitrogen until the day of the assays. Serum samples were stored at −20°C.

For this study, we selected 17 HIV^+^ children (median age of 12.9 years) and 12 HIV^−^ children (median age of 9.2 years). Patients were classified as responders (Rs) to the vaccine if they showed at least 4-fold increase in serum bactericidal antibody levels after vaccination. For HIV^+^ group and HIV^−^ group 10 (62%) and 6 (38%) individuals were classified as Rs, respectively.

### Bactericidal assay

Serum bactericidal antibody (SBA) titers were measured as previously described (5, 7), using human complement source.

### Polychromatic flow cytometry

#### Antibodies

Flow cytometry was performed using the following directly conjugated antibodies (clones): (1) BD Biosciences: CD3 H7APC (SK7 clone), CD45RA PE- Cy7 (L48), (2) BioLegend: CD3 BV650 (OKT3), IgM BV570 (MHM-88), PD-1 BV711 (EH12.2H7), CCR6 BV785 (G034E3), CD27 Alexa Fluor 594 (IA4CD27), CD27 BV605 (O323), CCR7 BV605 (G043H7), CCR7 APC (G043H7), CD8 BV650 (RPA-T8), ICOS Pac Blue (C398.4A), CD38 BV785 (HIT2); CXCR5 Alexa Fluor 488 (J252D4), CXCR3 PE (G025H7) (3) Invitrogen: CD4 PE-Cy5.5 (S3.5) and Aqua LIVE/DEAD® amine viability dye; (4) eBioscience: CXCR5 PE-CY7 (MU5UBEE); (5) Southern Biotech: IgD-PE; (6) Beckman Coulter: CD19 ECD (J3-119). The IgG (Alexa Fluor 680) and CD21 (PE-CY5.5) were conjugated in-house.

#### Phenotypic analysis

PBMCs were resuspended in RPMI 1640 (Invitrogen) supplemented with 10% fetal bovine serum, 2 mM L-glutamine, 100 U/mL penicillin and 100 ug /mL streptomycin (Invitrogen). 1 – 2 × 10^6^ PBMCs were incubated with Aqua viability dye and surface stained with titrated amounts of antibodies to panel (1): CD3, CD4, CD8, CD27, CD45RA, CCR7, PD-1, CCR6, CXCR3, CXCR5, and ICOS or (2) CD3, CD19, CD21, CD27, CD38, IgD, IgG, IgM, CXCR5, and CCR7. Cells were then washed in RPMI and fixed with 1% paraformaldehyde. Events were collected on a modified LSRII flow cytometer (BD Immunocytometry Systems). Electronic compensation was performed with antibody capture beads (BD Biosciences) stained separately with antibodies used in the test samples. Data were analyzed using FlowJo Version 9.6 (TreeStar, Ashland, OR).

#### Measurement of serum cytokines

Serum samples collected before vaccination (V1) were used to measure soluble CD14 (sCD14), using standard ELISA assays according to manufacturer's instructions (Quantikine® ELISA, R & D Systems, Minneapolis, MN, USA) and for the following cytokines: IL-4, IL-10, IL-21, TNF-α, and IFN-γ using the LuminexMap platform multiplex assay (EMD Millipore, Billerica, MA, USA). For CXCL13 chemokine we used the ProcartaPlex Human BLC) Simplex kit (eBioscience, Waltham, MA USA). The Luminex Platform was from Department of Periodontics, UERJ, Rio de Janeiro, Brazil.

#### Serum IgG, IgM, and IgA measurements

Total immunoglobulin concentrations of serum samples collected before vaccination (V1) were done by turbidimetry (Architect c4000, Abbott, Illinois, U.S.A.) according to manufacturer's instructions (BioSystems, Barcelona, Spain) at Immunology Laboratory, Pedro Ernesto University Hospital-UERJ, Rio de Janeiro, Brazil.

#### Statistical analysis

Flow cytometry data were analyzed using FlowJo software, version 7.6.4 (Tree StarInc., Ashland, OR). The levels of significance of the differences between groups were examined by, either the Mann-Whitney test (unpaired samples) or the Wilcoxon matched-pair test (paired samples), as nonparametric data were obtained. The correlation between different measurements of immune response was analyzed using Spearman rank test, after graph analyses. These analyses were performed with the GraphPad-Prism software, version 7 (GraphPad Software, Inc., USA).

## Results

### Circulating CD27^−^IgD^−^CD21^−^CD38^+^ (tissue-like exhausted B cells) are increased in HIV^+^ non-responders and negatively correlates with SBA

**Figure 1:**
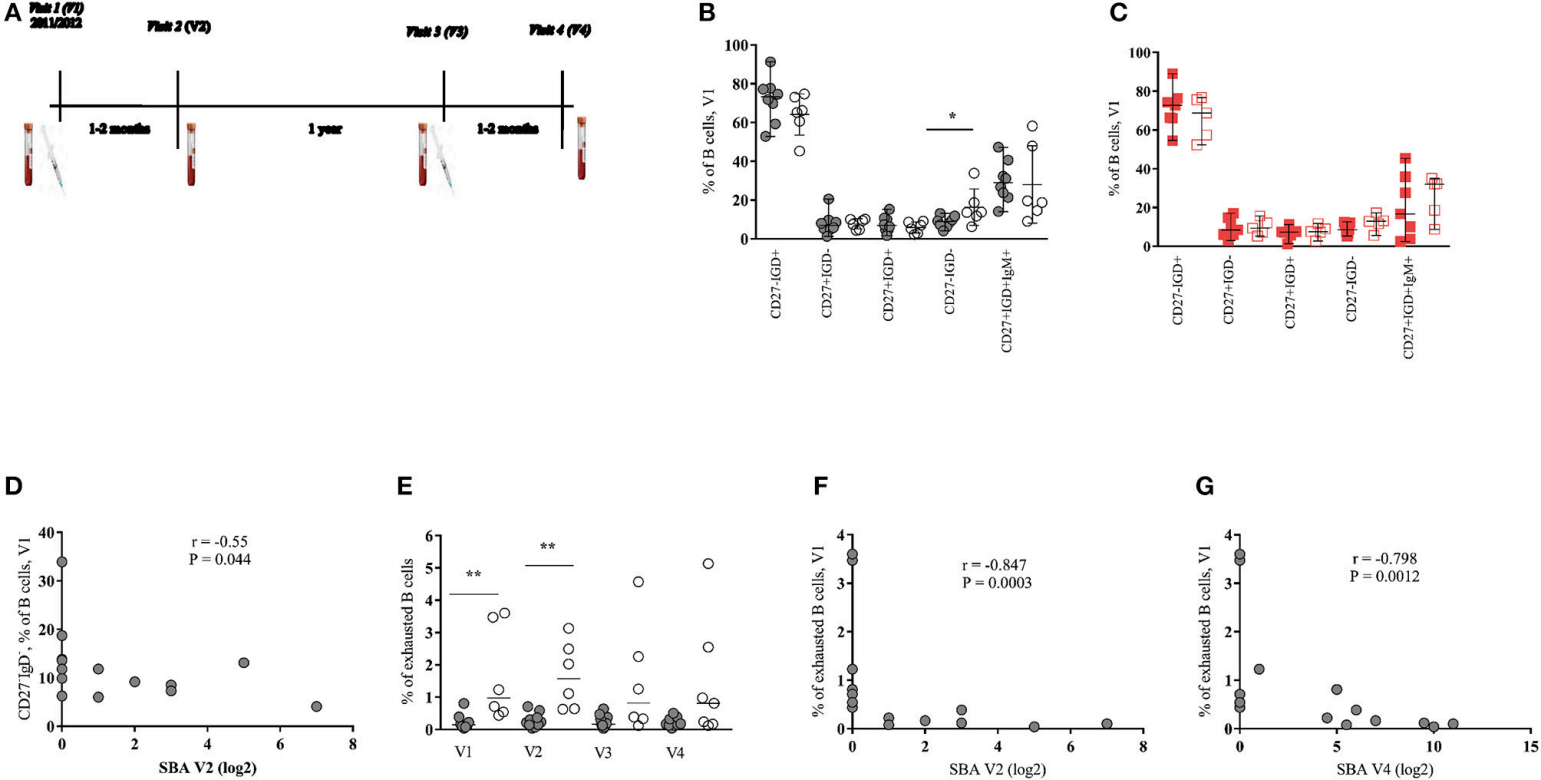
Baseline frequency of memory B cell subsets predict the response to MenC vaccination in HIV-infected young individuals. **(A)** Immunization schedule and blood sample collection of HIV-infected patients. HIV-uninfected received only one vaccine injection at visit 1 and had blood samples collected at visits 1 and 2 (V1 and V2). **(B,C)** Pooled data showing the relative frequencies of pre-vaccination (V1) B-cell populations, judged by the expression of CD27 and IgD, in **(B)** HIV^+^ responders (black closed circles) and non-responders (black open circles) and in **(C)** HIV^−^ responders (red closed squares) and non-responders (red open squares). **(D)** Serum bactericidal antibody (SBA) levels of HIV^+^ group post-one dose of vaccine (V2) inversely correlate with baseline frequencies of CD27^−^IgD^−^ B cells subset. **(E)** Exhausted B cells are increased in HIV^+^ non-responders compared with responders mainly at baseline and at V2. **(F,G)** SBA levels of HIV^+^ group after the first (V2) and the second dose of vaccine (V4) inversely correlate with frequencies of exhausted B cells, respectively. Lines represent the median with range values. *P*-values were calculated using Mann-Whitney test. Correlations were evaluated using a non-parametric Spearman rank correlation coefficient test. ^*^p < 0.05, ^**^p ≤ 0.01.

We have previously reported a significant negative correlation between the vaccine-induced bactericidal antibody responses and the frequency of circulating activated CD4 T cells in a Brazilian HIV-infected cohort aged 2–18 years (7). Here, we sought to extend these studies by analyzing bulk circulating CD4 and B cell population and its association with vaccine antibody response. The immunization schedule and time of blood collection according to patient visits at the pediatric clinic are shown in Figure 1A. Demographic, immunological and virological parameters for HIV-infected participants are shown in Table 1. No significant differences between HIV^+^ non-responders (NRs) and responders (Rs) were found when these parameters were analyzed, including time and length of cART (combination antiretroviral therapy) (Table 1). The median age of HIV^−^ Rs and NRs was 9.2 and 10.4 years, respectively. For HIV^−^ cohort, 55.5 and 71% of Rs and NRs, respectively, were female.

Circulating CD3^−^CD19^+^ B cell subsets, identified by the expression of surface markers, were analyzed as shown in Supplementary Data (Figure S1). First, the relative frequency of subsets defined based on the expression of CD27 and IgD molecules were determined. For HIV^+^ group, a significant difference (*p* = 0.032) was found only for the baseline frequency of CD27^−^IgD^−^ B cell subset between R and NR groups (Figure 1B and Figures S2A–C). No differences, however, were observed when the frequencies of B cell subsets were analyzed in the HIV^−^ group (Figure 1C and Figure S2D). Interestingly, a significant negative correlation (*r* = −0.55, *p* = 0.044) between the baseline (V1) frequency of CD27^−^IgD^−^ B cells and SBA measured after one dose of vaccine (V2) was found (Figure 1D). A similar picture was seen when we considered SBA after two doses (V4) of vaccine (*r* = −0.53, *p* = 0.054, data not shown). Contrary to HIV-infected group, no correlation between baseline CD27^−^IgD^−^ B cells and SBA was found for the HIV^−^ group (data not shown).

Decreased expression of CD21 and increased expression of CD38 is associated with activation and terminal B cell differentiation in HIV infection (16, 17). Therefore, we sought to analyze the expression of CD21 and CD38 on CD27^−^IgD^−^ and CD27^+^IgD^−^ (switched memory) B cell populations. A higher baseline (*p* = 0.005) and V2 (*p* = 0.001) frequency of CD27^−^IgD^−^CD21^−^CD38^+^ B cells (hereafter described as exhausted B cells), in HIV^+^ NRs compared to Rs was found (Figure 1E). Significant inverse correlations between baseline exhausted B cells and SBA after one (V2) and two (V4) doses of vaccine were also found in the HIV-infected group (Figures 1F,G). For HIV-uninfected cohort, we observed a trend for higher baseline frequencies of exhausted B cells at V1 and V2 (Figure S2E). Regarding the switched B cells (CD27^+^IgD^−^), HIV^+^ NRs showed higher frequency of CD38^+^CD21^+^ cells than HIV^+^ Rs at all time points studied but V3 (Figures S3A–D). A trend for high levels of CD27^+^IgD^−^CD21^−^CD38^+^ B cells (short-lived plasmablasts, hereafter described as plasmablasts) was also seen in NRs, especially at V2 (Figure S3B). A significant negative correlation was found between the baseline frequency of CD27^+^IgD^−^CD21^+^CD38^+^ B cells and SBA at V2 and V4 (Figures S3E,F) as well as between the frequency of that B cell phenotype at V3 and SBA at V4 (Figure S3G). A trend (*p* = 0.073) for higher frequency of plasmablasts in NRs compared to Rs was found in HIV^−^ individuals (Figure S3H).

For both HIV^+^ and HIV^−^ cohorts, we did not detect significant differences in the frequency of IgG^+^ or IgM^+^ switched B cells (data not shown) at any time point studied. Furthermore, no differences were found when the expression of CXCR5 and CCR7 on CD27^−^IgD^−^ and CD27^+^IgD^−^ B cells, between Rs and NRs, from both cohorts were analyzed (data not shown). Our data indicate that exhausted B cells and CD27^+^IgD^−^CD21^+^CD38^+^ B cells as well as plasmablasts are increased during treated HIV infection and negatively associated with the MCC vaccine induced antibody response.

### Baseline frequency of activated peripheral TFH cells inversely correlates with vaccine induced antibodies

**Figure 2:**
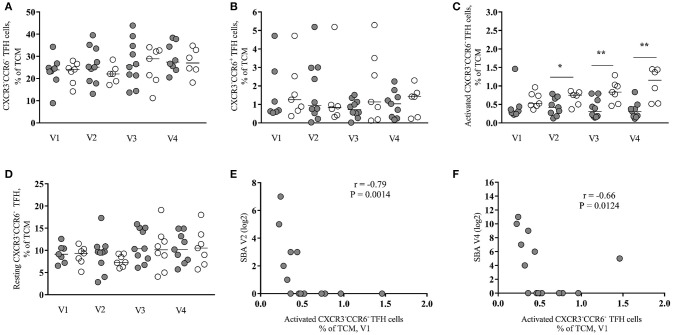
Baseline frequency of activated peripheral TFH cells inversely correlates with vaccine antibody response in HIV-infected individuals. **(A)** Frequency of CXCR3^−^CCR6^−^ peripheral TFH cells in HIV^+^ responders (closed circles) and non-responders (open circles) pre- (V1 and V3) and post-vaccination (V2 and V4). **(B)** Frequency of CXCR3^−^CCR6^+^ peripheral TFH cells. **(C)** Frequency of activated (CCR7^−^ PD1^++^ICOS^+^) CXCR3^−^CCR6^−^ peripheral TFH cells. **(D)** Frequency of resting (CCR7^+^PD1^−/+^ICOS^−^) CXCR3^−^CCR6^−^ peripheral TFH cells. **(E,F)** The frequency of baseline (V1) activated CXCR3^−^CCR6^−^ peripheral TFH cells negatively correlated with SBA induced by one vaccine injection (V2) (*r* = −0.79, *P* = 0.0014) and with SBA induced by two vaccine injections (V4) (*r* = −0.66, *P* = 0.0124). Lines represent the median values. *P*-values were calculated using Mann-Whitney test. Correlations were evaluated using a non-parametric Spearman rank correlation coefficient test. ^*^p < 0.05, ^**^p ≤ 0.01.

Recently, several studies have been focused on the analysis of circulating CD4 subsets with increased capacity for *in vitro* B cell help as surrogates of the development of TFH cells in the secondary lymphoid organs (8, 18, 19). We investigated the dynamics of peripheral TFH subsets based on the expression of CXCR5, CCR6 and CXCR3 (9, 10), the gating scheme is shown in Figure S4. A significantly higher frequency of CXCR3^−^CCR6^−^ (median values: 22.1–28.9% from V1 to V4) compared to CXCR3^−^CCR6^+^ (median values: 0.68–1.41%) peripheral TFH cells was found in the HIV-infected group at all visit times (Figures 2A,B). No significant differences were found between Rs and NRs, when these populations were analyzed in all samples tested (Figures 2A,B). Similar data were observed for the HIV-non-infected group (Figures S5A,B). The expression of CCR7, PD-1 and ICOS receptors (CCR7^−^PD1^++^ICOS^+^ and CCR7^+^PD1^−/+^ICOS^−^subsets) was used as a surrogate for the activation of these cells. We saw higher frequency of activated (CCR7^−^PD1^++^ICOS^+^) CXCR3^−^CCR6^−^ cells for NRs compared to Rs for all time points reaching statistical significance at V2, V3, and V4 (V1 = 0.053) (Figure 2C). In contrast to activated peripheral TFH, similar frequencies of “resting” (CCR7^+^PD1^−/+^ICOS^−^) CXCR3^−^CCR6^−^ peripheral TFH were found for both group of vaccinees (Figure 2D). We did not observe a consistent pattern for activated or resting CXCR3^−^CCR6^+^ peripheral TFH cells (data not shown**)**. A similar trend, although no significant, was found when CXCR3^−^CCR6^−^ activated peripheral TFH cells from HIV-non-infected individuals were analyzed (Figures S5C,D). Next, the association between baseline CXCR3^−^CCR6^−^ activated peripheral TFH cells and the SBA responses was analyzed. The baseline frequency of activated peripheral TFH cells negatively correlated with SBA at V2 (*r* = −0.79, *p* = 0.0014) and V4 (*r* = −0.66, *p* = 0.0124) (Figures 2E,F). The same pattern of association was registered for correlation analysis between the frequency of activated peripheral TFH cells at V2 and V3 and SBA at V4 (data not shown). A similar trend, although not significant, was found when the baseline frequency of activated peripheral TFH cells and SBA in HIV-non-infected individuals was analyzed (data not shown).

**Figure 3:**
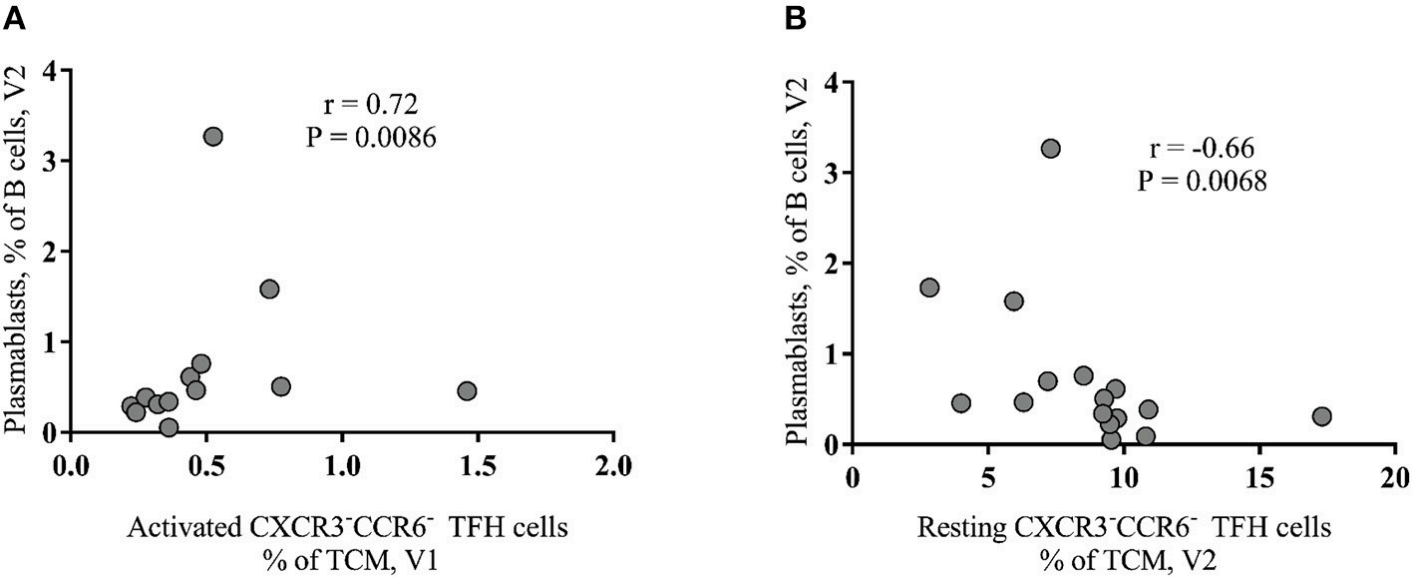
Baseline activated peripheral CXCR3^−^CCR6^−^ TFH cells correlate with plasmablasts after vaccination of HIV^+^ group. **(A)** A positive association between activated peripheral TFH cells at V1 and plasmablasts (CD27^+^IgD^−^CD21^−^CD38^+^ B cells) at V2. **(B)** The frequency of resting peripheral TFH cells, detected after the first vaccination (V2), negatively correlated (*r* = −0.66, *P* = 0.0068) with plasmablasts.

Next, we asked whether there is any association between B cell subsets and peripheral TFH cells. We found a significant positive correlation between the baseline frequency of activated peripheral TFH cells and the frequency of plasmablasts at V2 (*r* = 0.72, *p* = 0.0086) (Figure 3A) while CXCR3^−^CCR6^−^ resting peripheral TFH cells at V2 negatively correlated with short-lived plasmablasts at V2 (*r* = −0.66, *p* = 0.0068) (Figure 3B). No significant correlations were found when similar cell populations from HIV-non-infected individuals were analyzed (data not shown). Regarding the CXCR3^−^CCR6^+^ peripheral TFH cells measured in HIV^+^ cohort, we found a significant inverse correlation between both activated (*r* = −0.756, *p* = 0.041) and resting (*r* = −0.829, *p* = 0.0167) phenotypes measured before the second dose of vaccine (V3) and SBA levels post-second vaccination (V4) (data not shown). Taken together, our data indicate that the baseline frequency of activated CXCR3^−^CCR6^−^ peripheral TFH cells is a negative correlate for the bactericidal antibody response to MCC vaccine in cART treated young individuals.

### Baseline serum levels of IL-4 correlate with vaccine-elicited protective antibody titers

**Figure 4:**
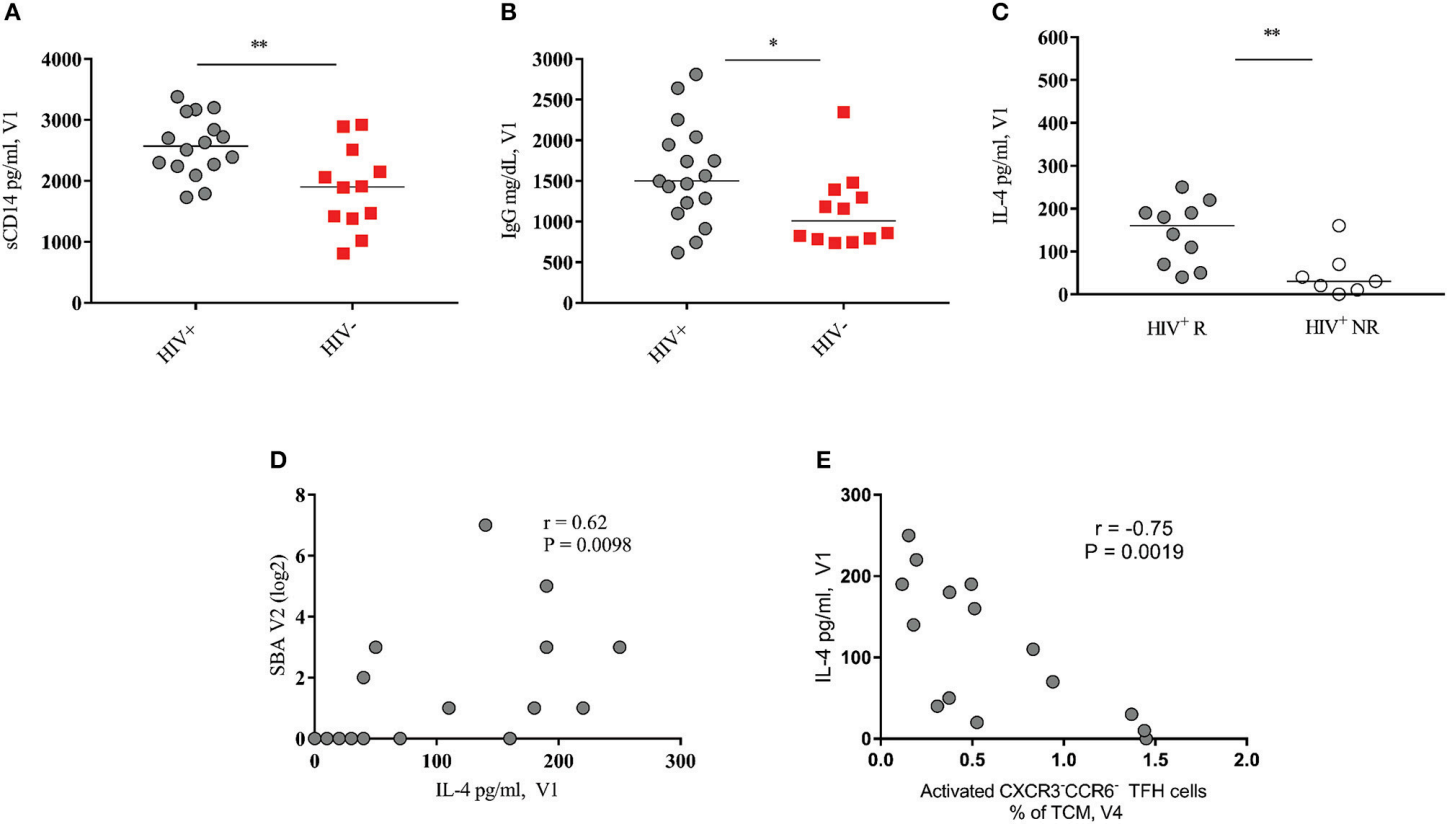
Baseline serum levels of IL-4 correlate with SBA response in HIV^+^ vaccinees. **(A,B)** Pooled data showing soluble CD14 and IgG levels in HIV^+^ (black circles) and HIV^−^ (red squares) vaccinees, respectively. **(C)** Serum IL-4 levels of HIV^+^ responders (closed circles) are significantly higher than of non-responders (open circles). **(D)** Baseline HIV^+^ blood IL-4 levels positively correlate with SBA at V2. **(E)** Baseline HIV^+^ blood IL-4 levels negatively correlated with activated peripheral CXCR3^−^CCR6^−^ TFH cells at V4. Lines represent the median values. *P*-values were calculated using Mann-Whitney test. Correlations were evaluated using a non-parametric Spearman rank correlation coefficient test. ^*^p < 0.05, ^**^p ≤ 0.01.

As expected (20), significantly higher baseline (V1) serum sCD14 (*p* = 0.0087) were found in HIV-infected compared to non-infected individuals (Figure 4A). TNF-α levels were similar for HIV-infected or non-infected, for both, Rs and NRs (data not shown). INF-γ was detected in sera of a few individuals (data not shown) while no IL-10 or IL-21 was detected in any serum sample of our cohort. Total IgG levels (V1) were found significantly higher in HIV-infected compared to non-infected individuals (*p* = 0.0339) (Figure 4B). However, no difference in baseline IgG or sCD14 concentration was found between HIV^+^ Rs and NRs (data not shown). Furthermore, no difference was found in the levels of blood (V1) IgM or IgA between the two cohorts (data not shown).

Next, the circulating levels of IL-4 and CXCL-13, cytokines involved in the follicular B cell response development (8, 9, 18) were analyzed. IL-4 levels were significantly higher in Rs compared to NRs selectively in the HIV-infected group (*p* = 0.006) (Figure 4C, Figure S6C shows IL-4 levels for HIV^−^ cohort). We found a significant correlation between baseline (V1) serum levels of IL-4 and vaccine-induced antibody titers at V2 (*r* = 0.62, *p* = 0.0098) (Figure 4D) and V4 (*r* = 0.52, *p* = 0.033, Figure S6B). In contrast, a significant negative association was found between baseline IL-4 levels and the frequency of activated peripheral TFH cells after the second immunization (V4) (Figure 4E). Regarding CXCL-13, no significant differences were found between Rs and NRs for both cohorts (Figures S6A,D). Although not significant, HIV-non-infected vaccinees showed lower levels of CXCL-13 compared to HIV-infected ones (Figures S6A,D). There was no correlation between baseline circulating levels of IgG and CXCL-13 in HIV^+^ subjects. However, a positive association was found between baseline levels of TNF-α and CXCL-13 for HIV^+^ (*r* = 0.51, *p* = 0.052) and HIV^−^ cohort (*r* = 0.62, *p* = 0.035) (data not shown). Therefore, IL-4 and CXCL-13 blood levels are differentially correlated with the development of the vaccine-induced B cell responses in cART treated young individuals.

## Discussion

MCC vaccine has been shown to be safe and immunogenic in many high-risk populations, with results depending on the degree of immunosuppression and/or immune hyperactivation (7, 21). Suboptimal immune responses to immunization with MCC vaccine results in poor serum bactericidal antibody response that correlates with inadequate protection (22). Different of polysaccharide vaccines, conjugate vaccines induce a T-cell dependent antibody response with predominance of IgG antibodies (21, 23). We took advantage of a cohort of HIV^+^ children/adolescents, with or without response to MCC vaccine (5–7), to further investigate possible associations between relevant circulating immune cell populations and vaccine antibody response. Our previous work has revealed that activation of circulating CD4 T cell, judged by the expression of CD38, HLA-DR, and CCR5, was negatively associated with MCC induced responses in this cohort (7).

We described here a significant higher baseline frequency of CD27^−^IgD^−^ B cells in HIV^+^ NRs compared to HIV^+^ Rs, with baseline CD27^−^IgD^−^ B cell frequency negatively correlating with vaccine antibody response. Furthermore, we found a negative correlation between the baseline frequency of exhausted B cells and vaccine antibody response for HIV^+^ cohort. Noteworthy, the frequency of exhausted B cell subset was consistently higher in NRs compared to Rs. In contrast, HIV-uninfected group had no significant differences in the frequency of exhausted B cell subset before or after vaccination. Therefore, the generalized activation of B cells, evident by the elevated blood total IgG level, accompanied by a skewed differentiation to less functional tissue-liked exhausted B cells and short-lived plasmablasts is associated with non-responsiveness to MCC vaccine in cART HIV^+^ children.

Several recent reports have been focused on the analysis of circulating counterparts of follicular TFH (peripheral TFH) (8, 10–12). We found a consistently higher frequency of activated (CCR7^−^PD1^++^ICOS^+^ CXCR3^−^CCR6^−^ TCM) peripheral TFH cells in HIV infected NRs compared to Rs. Furthermore, this population was positively correlated with tissue like exhausted B cells but showed a negative correlation with SBA. CXCR3^−^CCR6^−^ TFH cells express a Th2-like rather than a Th1- or Th17-like functional profile (9). However, T follicular regulatory cells could also express the same surface markers as described here for TFH cells (24). Due to limited cell availability, we were not able to perform functional assays to address the specific functional profile of the populations under investigation and elucidate possible mechanisms contributing to the negative effect of activated peripheral TFH cells on B cell response. However, we have observed a significant negative correlation between CD4 TCM expressing several activation markers (PD1, TIGIT, HLA-DR, and CD38) with SBA in our HIV^+^ cohort (unpublished data). Therefore, it is possible that peripheral TFH cells express a “hyperactive” profile in NRs associated with impaired cell-cell interactions and possibly diminished help for antibody responses (19, 25, 26). In line with this, we found significantly higher levels of sCD14 in the HIV^+^ compared to HIV^−^ group, indicating that despite no significant differences in duration or the age of initiation of cART, there was still a higher immune activation in HIV^+^ individuals. Although the levels of circulating CXCL-13, a chemokine that has been described as an indicator of germinal center reactivity (27), did not differ between Rs and NRs, we found that IL-4, an important cytokine for germinal center B cell development (28, 29), was significantly upregulated in Rs compared to NRs. Furthermore, we showed here a positive correlation between baseline IL-4 levels and vaccine antibody response while it was negatively associated with the frequency of peripheral activated TFH cells. Despite their role in germinal center response, this profile could reflect the different sources, in addition to TFH cells (29, 30), for these two cytokines.

Expression of CXCR5 has been widely used for the identification of peripheral TFH cells (8, 9). This CD4 T cell pool, however, is highly heterogeneous concerning the profile of cytokine production and the capacity for *in vitro* B cell help (8, 18). Whether peripheral TFH cells originate from lymph node germinal center TFH cells or represent a transient pre-TFH or a follicular, non-germinal center TFH population is not well understood (18, 19, 25). Their increased frequency found in NRs implies that higher activation/differentiation of CD4 T cells within the lymph node is not necessarily associated with induction of vaccine antibody response. Particularly in chronically HIV-infected patients, where other factors including lymph node structure damage, follicular hyperplasia and lysis and loss of follicular dendritic cell network (31, 32) could play a major role for the development of vaccine-induced response. Furthermore, cART results in low/undetectable viremia and lower immune activation, although not to levels found in HIV^−^ individuals, in blood (33) and lymph node level (34).

Given the limited, if any, access to lymph nodes from vaccine clinical trials the identification of biomarkers or blood signatures for monitoring vaccine efficacy is of great importance. An association between vaccine responses and peripheral TFH cells (12, 35–37) or CXCL-13 (27, 38, 39), a surrogate of germinal center reactivity (27) has been previously shown. We have recently reported a negative correlation between baseline levels of circulating CXCL-13 and hepatitis B vaccine responses in treated HIV infected adults (39). However, our current data show that this is not the case for cART infected young individuals receiving MCC vaccine. Whether this is related to the nature of the immunogen or other biological factors like aging is not known. Further studies are needed to confirm whether the simultaneous analysis of circulating CXCL-13, IL-4 and activated peripheral TFH cells could be used as a “biomarker” for monitoring vaccine efficiency. Furthermore, whether the capacity for response to specific immunogens reflects the overall quality of host immune responses to HIV (39) is not clear. A direct comparison between Th1, Th2, and lymph node TFH specific responses and immunogen-specific B cell responses would provide valuable information regarding this. To this end, the use of non-human primate models, where the parallel analysis of circulating and lymph node dynamics is feasible, is of great importance.

## Ethics statement

All participant's parents or legal guardians provided written informed consent, as well as the participants who were aware of their HIV-infection status. The study was approved by the IPPMG Institutional Review Board (IRB, number 24/09) and Brazilian Ministry of Health Ethics Comission (*Comissão Nacional de Ética em 000Pesquisa*, CONEP, number 15578). Details about the inclusion and exclusion criteria, IRB and participant's consent were previously published (5).

## Author contributions

LM performed the experiments and analysis and wrote the manuscript. GS, MF, BC, and WP-M performed experiments. AF, BF, DB, and CH coordinated the recruitment and immunization of patients, the collection of biological specimens and patient follow-up. CP designed, analyzed experiments and wrote the manuscript. RK provided critical review of data and manuscript. All authors read and approved the final version of the manuscript.

### Conflict of interest statement

The authors declare that the research was conducted in the absence of any commercial or financial relationships that could be construed as a potential conflict of interest.
