# Supplementary material for: Baseline Circulating Activated TFH and Tissue-Like Exhausted B Cells Negatively Correlate With Meningococcal C Conjugate Vaccine Induced Antibodies in HIV-Infected Individuals
Source: Front Immunol. 2018 Oct 29;9:2500. doi: 10.3389/fimmu.2018.02500 (PMC6215828; doi:10.3389/fimmu.2018.02500)
Supplement: Supplementary file 1 [file Presentation_1.pdf]

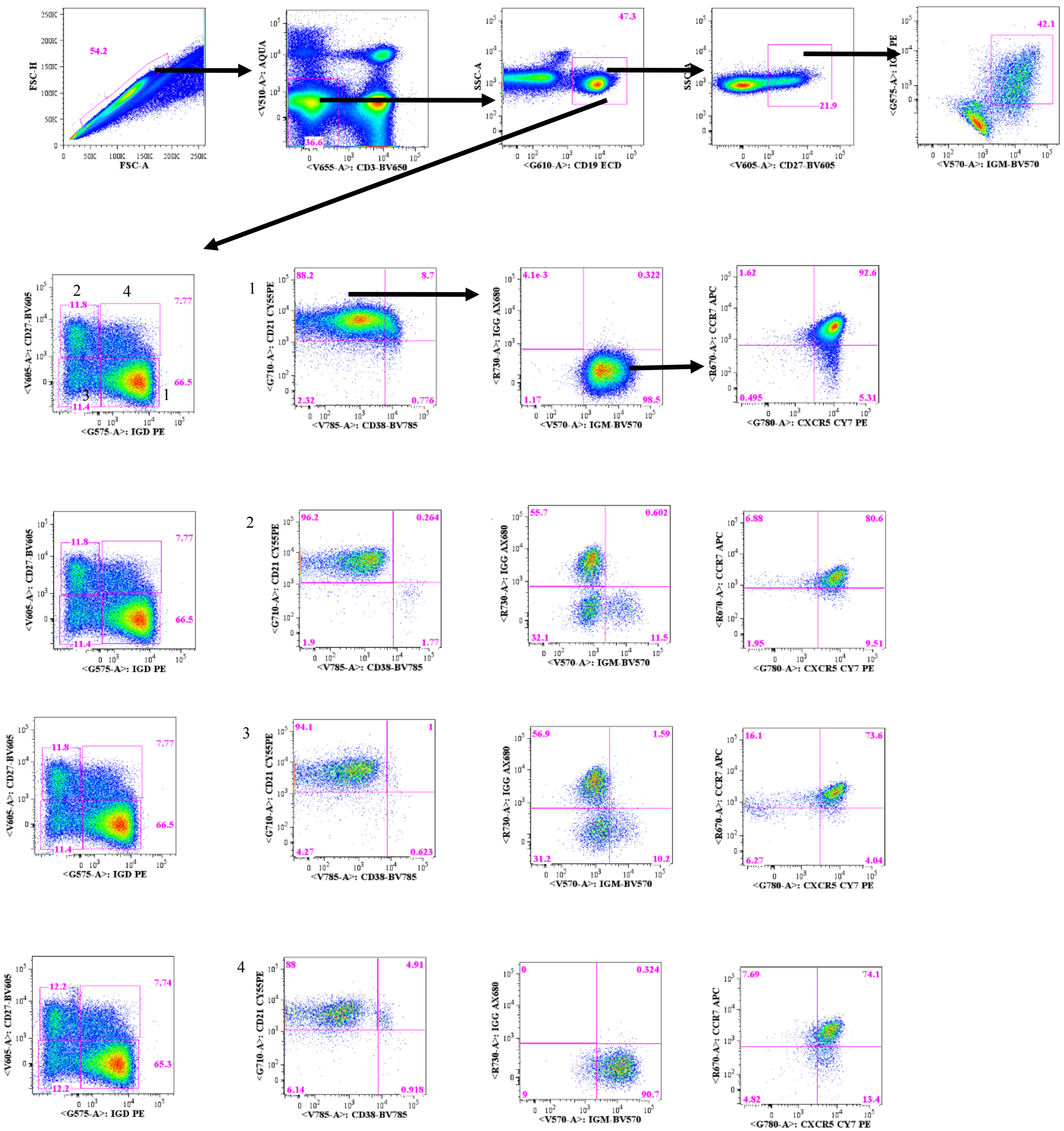

Supplemental Figure 1 – B cells ($CD3^{-}CD19^{+}$) strategy of analysis of Flow Cytometry data from one representative experiment with PBMC sample of HIV-uninfected individual.

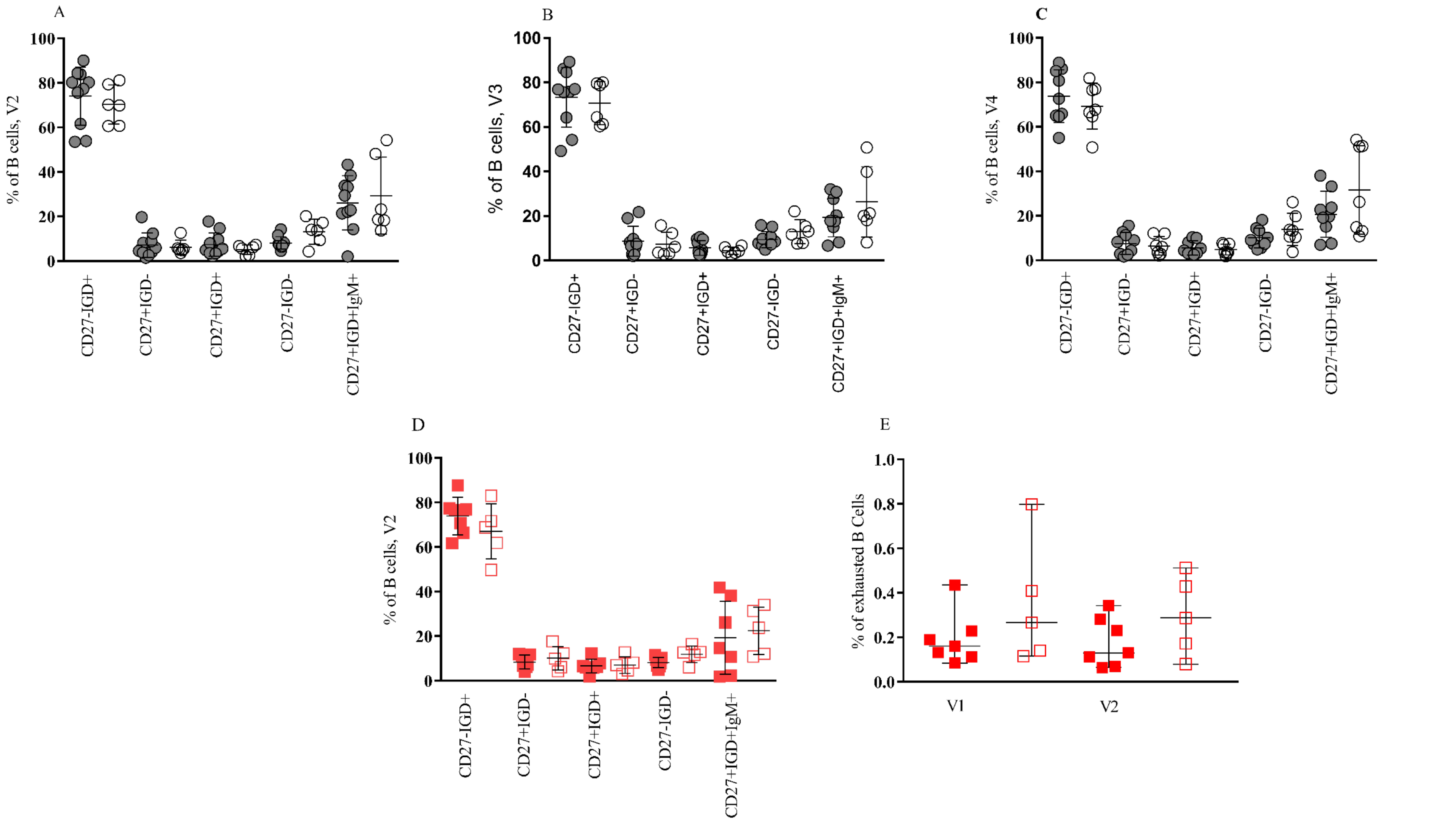

Supplemental Figure 2 - B cell subsets based on CD27 and IgD expression. (A - C) Pooled data showing the relative frequencies of B-cell populations in HIV^{+} responders (black closed circles) and non-responders (black open circles) after the first vaccination (V2), before the second dose of vaccine (V3) and post-second dose of vaccine (V4). **(D)** relative frequencies of B-cell populations in HIV $^{-}$ responders (red closed squares) and non-responders (red open squares) post-vaccination (V2). **(E)** A trend for higher frequency of exhausted B cells (CD27^{-}IgD^{-}CD21^{-}CD38^{+} was seen in HIV^{-} NRs (red open squares) compared to Rs (red closed squares). Line represent the median with range values. P-values were calculated using Mann-Whitney test.

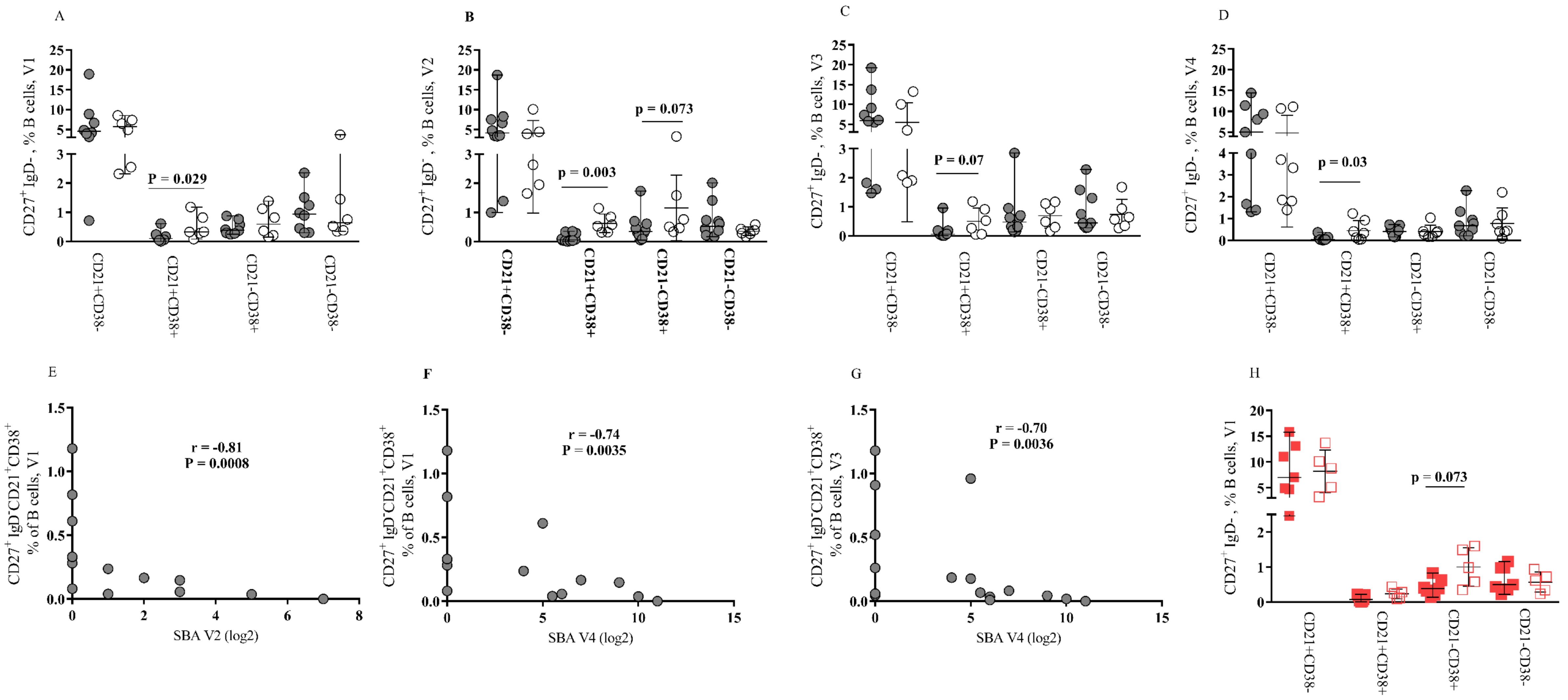

Supplemental Figure 3 - Frequency of switched B cells expressing CD21 and CD38 are higher in HIV$^+$ NRs and negatively correlate with SBA. (A - D) Pooled data showing the relative frequencies of CD27$^+$IgD$^-$ B-cells expressing different combinations of CD21 and CD38 molecules in HIV$^+$ responders (black closed circles) and non-responders (black open circles) at baseline (V1), after the first vaccination (V2), before the second dose of vaccine (V3) and post-second dose of vaccine (V4). **(E-F)** SBA levels of HIV$^+$ group after the first (V2) and the second dose of vaccine (V4) inversely correlate with frequencies of CD27$^+$IgD$^-$CD21$^+$CD38$^+$ B cells, respectively. **(G)** CD27$^+$IgD$^-$CD21$^+$CD38$^+$ B cell frequency before the second vaccination (V3) negatively correlated with SBA post-vaccination (V4). **(H)** A trend for higher frequency of short-lived plasmablasts (CD27$^+$IgD$^-$CD21$^-$CD38$^+$) was seen in HIV$^-$ NRs (red open squares) compared to Rs (red closed squares). Lines represent the median with range values. P-values were calculated using Mann-Whitney test. Correlations were evaluated using a Spearman rank correlation coefficient test. `

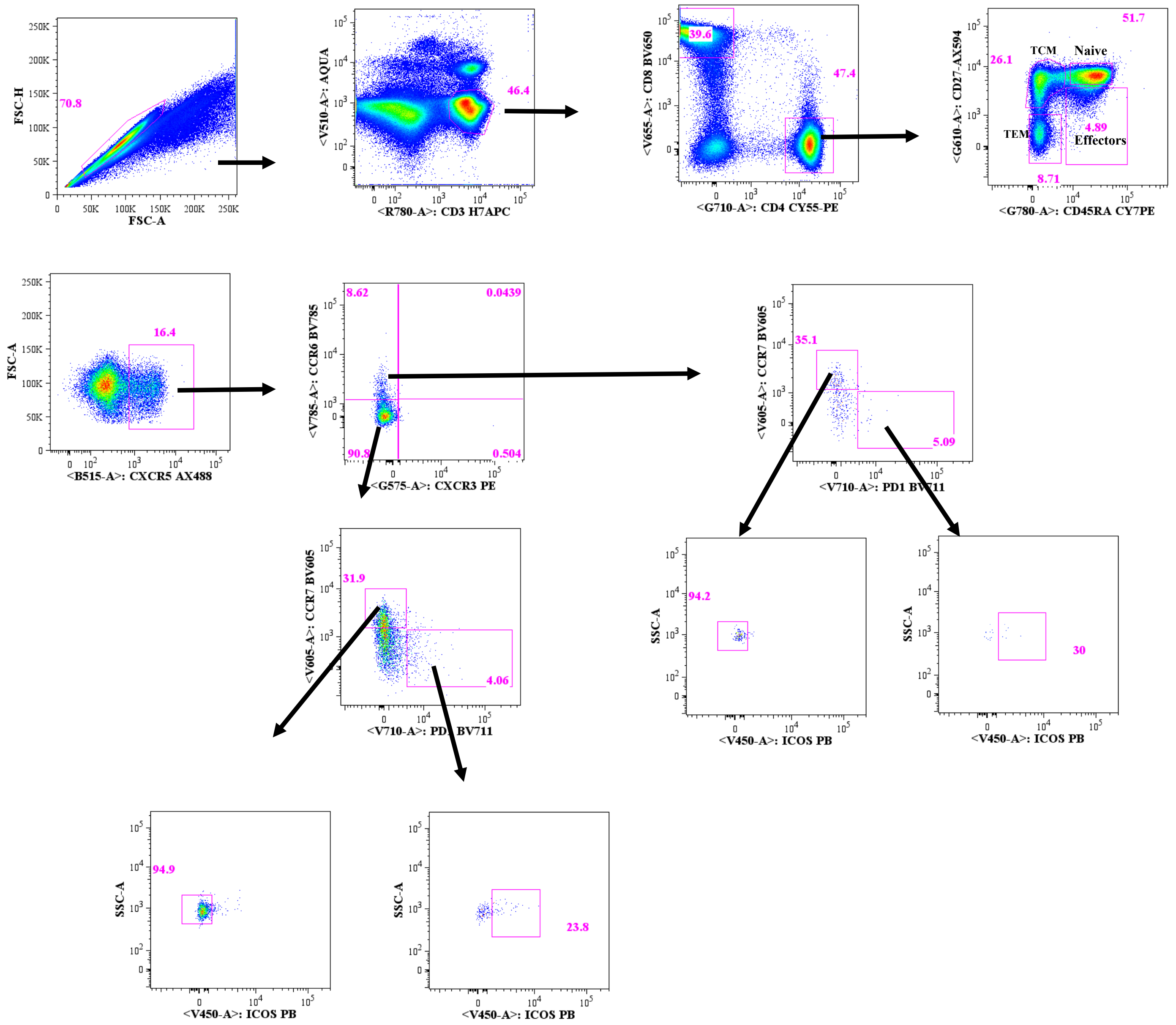

Supplemental Figure 4 – Peripheral TFH cells strategy of analysis of Flow Cytometry data from one representative experiment with PBMC sample of HIV-uninfected individual.

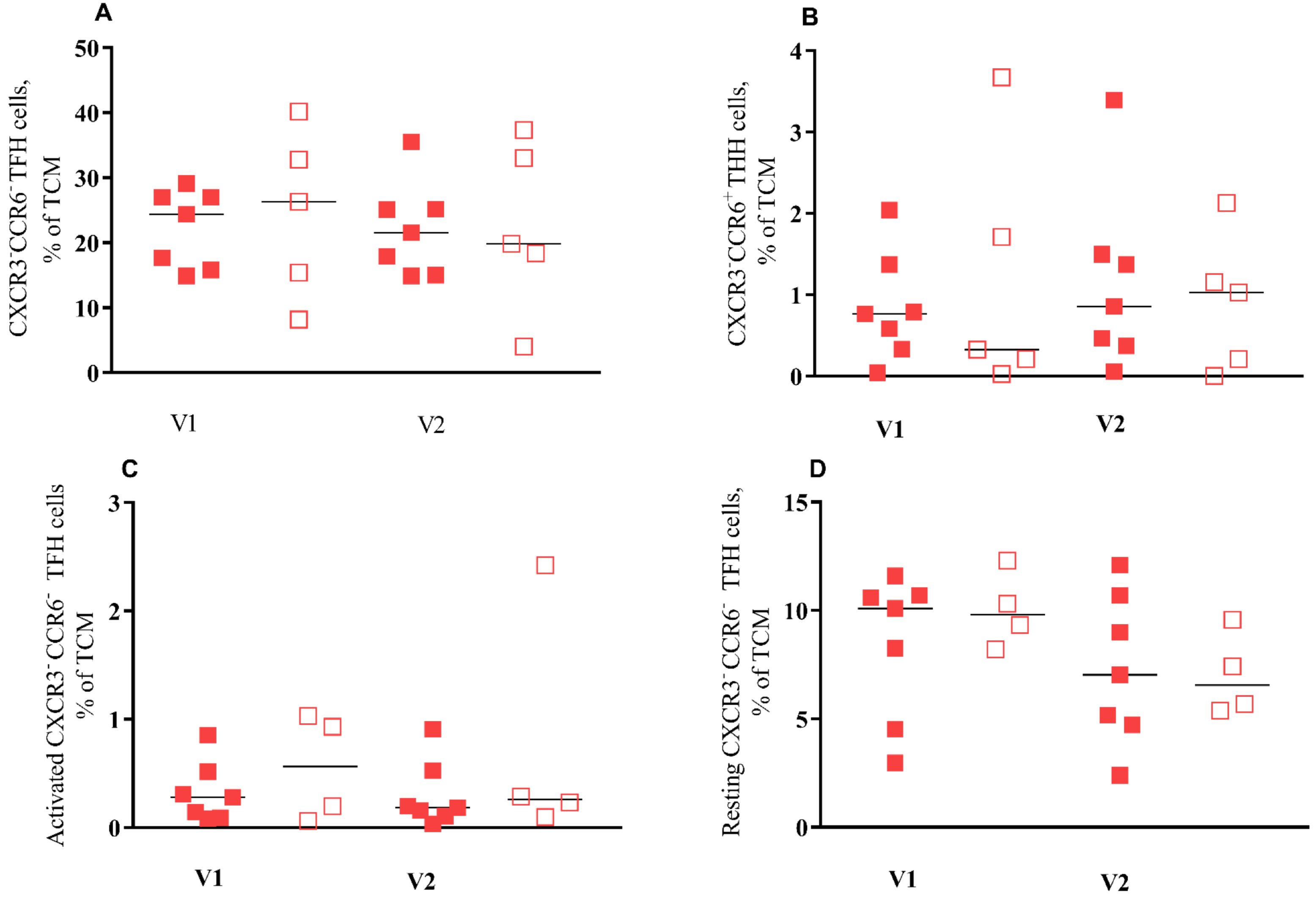

Supplemental Figure 5 - Circulating TFH cells in HIV$^-$ cohort. (A) Frequency of CXCR3$^-$CCR6$^-$ peripheral TFH cells in HIV$^-$ responders (closed squares) and non-responders (open squares) pre- (V1) and post-vaccination (V2). **(B)** Frequency of CXCR3$^-$CCR6$^+$ peripheral TFH cells. **(C)** Frequency of activated (CCR7$^-$PD1^{++}ICOS$^+$) peripheral CXCR3$^-$CCR6$^-$ TFH cells. **(D)** Frequency of resting (CCR7$^+$PD1$^{+/-}$ICOS$^-$) peripheral CXCR3$^-$CCR6$^-$ TFH cells.

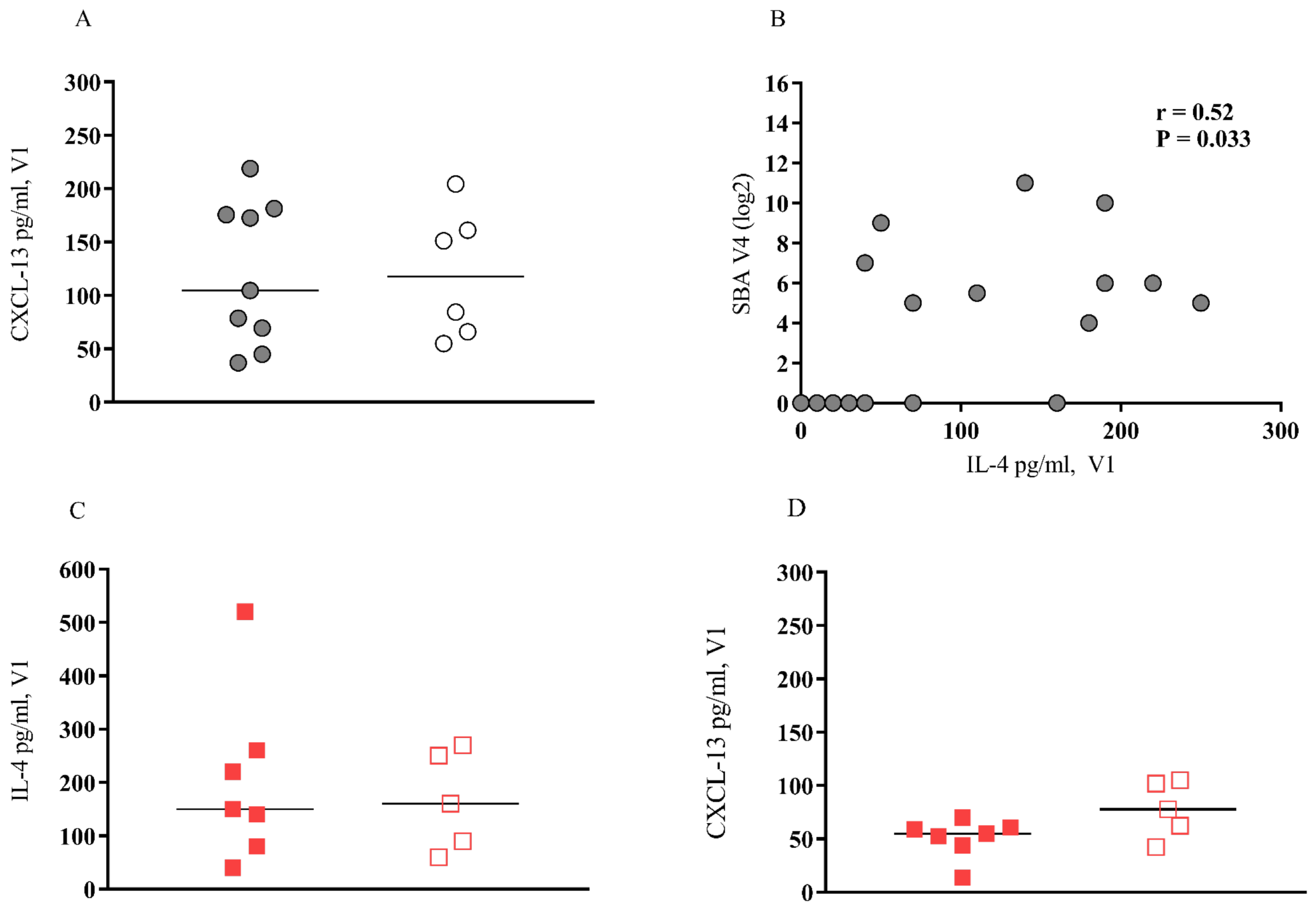

Supplemental Figure 6 – HIV⁺ cohort shows similar levels of CXCL-13 for R and NRs. **(A)** Plasma levels of CXCL13 in HIV⁺ responders (black closed circles) and non-responders (black open circles) at baseline **(B)** Baseline HIV⁺ blood IL-4 levels positively correlate with SBA at V4. **(C-D)** Plasma levels of IL-4 and CXCL-13 in HIV⁻ responders (red closed squares) and non-responders (red open squares), respectively. Lines represent the median values. P-values were calculated using Mann-Whitney test. Correlations were evaluated using a Spearman rank correlation coefficient test.
